# Arts-Based Compassion Skills Training (ABCST): Channelling Compassion Focused Therapy Through Visual Arts for Australia’s Indigenous Peoples

**DOI:** 10.3389/fpsyg.2020.568561

**Published:** 2020-12-16

**Authors:** James Bennett-Levy, Natalie Roxburgh, Lia Hibner, Sunita Bala, Stacey Edwards, Kate Lucre, Georgina Cohen, Dwayne O’Connor, Sharmaine Keogh, Paul Gilbert

**Affiliations:** ^1^University Centre for Rural Health, The University of Sydney, Lismore, NSW, Australia; ^2^RealArtWorks, Lismore, NSW, Australia; ^3^Department of Psychology, Charles Sturt University, Bathurst, NSW, Australia; ^4^Birmingham and Solihull Mental Health NHS Foundation Trust, Birmingham, United Kingdom; ^5^Rekindling the Spirit, Lismore, NSW, Australia; ^6^Centre for Compassion Research and Training, University of Derby, Derby, United Kingdom

**Keywords:** compassion focused therapy, Indigenous, Aboriginal, art, self-compassion, compassion, group-based treatment

## Abstract

The last 20 years have seen the development of a new form of therapy, compassion focused therapy (CFT). Although CFT has a growing evidence base, there have been few studies of CFT outside of an Anglo-European cultural context. In this paper, we ask: Might a CFT-based approach be of value for Indigenous Australians? If so, what kind of cultural adaptations might be needed? We report the findings from a pilot study of an arts-based compassion skills training (ABCST) group, in which usual CFT group processes were significantly adapted to meet the needs of Indigenous Australians. At face value, CFT appeared to be a promising approach to enhancing the social and emotional wellbeing of Australia’s Indigenous peoples. However, despite initial consultations with Indigenous health professionals, the first attempts to offer a more conventional group-based CFT to Indigenous clients were largely unsuccessful. Following a review and advice from two Indigenous clients, we combined elements of CFT with visual arts to develop a new approach, “arts-based compassion skills training” (ABCST). This paper reports an evaluation of this pilot ABCST group. The group had 6 × 4 h sessions of ABCST, facilitated by two psychologists (1 Indigenous, 1 non-Indigenous) and two artists (1 Indigenous, 1 non-Indigenous). There were 10 participants, who attended between 2 and 6 sessions: five were clients, five were health professionals. Between 1 and 3 months later, six of the participants (2 clients, 4 health professionals) were interviewed. Qualitative analysis of interview data identified that two key processes—creating a positive group atmosphere and channeling compassion skills training through the medium of visual arts—led to four positive outcomes for participants: planting the seeds of new understandings, embodying the skills of compassion, strengthening relationships with others, and evolving a more self-compassionate relationship. We suggest that the preliminary results are sufficiently encouraging to warrant further development of ABCST in Indigenous communities.

## Introduction

The strength and resilience of Australia’s Aboriginal and Torres Strait Islander peoples (henceforth respectfully termed Indigenous Australians or Indigenous peoples) can be measured by the fact that this may be the oldest culture on earth with continuous occupation of Australia for at least 60,000 years (Malaspinas et al., 2016; Florin et al., 2020). That strength and resilience remains a key feature of Indigenous Australians in the twenty-first century, in the face of the massive disruption, exploitation and trauma which have resulted from European colonization (Australian Human Rights Commission, 1997; Dudgeon et al., 2014; Cronin et al., 2019).

Fundamental to Indigenous Australians’ conception of strength, resilience and health is the idea of connectivity across multiple domains, grounded in the concept of Social and Emotional Wellbeing (SEWB) (Gee et al., 2014; Butler et al., 2019). The SEWB model suggests that optimal health and wellbeing is associated with strong connections across seven domains: connection to body, mind and emotions, family and kinship, community, culture, land, and spirit (Gee et al., 2014). Conversely when these connections are disrupted, Indigenous ill health is a likely outcome (Dudgeon et al., 2014). It is evident that this Indigenous worldview is more holistic and encompassing that the western concept of “mental health.”

Since the first European colonization in 1788, the history of Indigenous Australians has been marked by genocide, transgenerational trauma, abuse, marginalization and discrimination. For instance, the experiences of the Stolen Generation have led to mass disconnections across all areas of SEWB, particularly connection to family, country and culture (Australian Human Rights Commission, 1997). This transgenerational history of trauma, abuse and racism has led to huge disparities in present day socio-economic status, education and health compared with the non-Indigenous population of Australia (Dudgeon et al., 2017; Brockman and Dudgeon, 2019). Relatedly, on all measures of psychological wellbeing, mental health, and suicide, Australia’s Indigenous peoples have far poorer outcomes, with three to six times the mental distress of non-Indigenous Australians, far higher suicide rates particularly amongst Indigenous youth, and destructive levels of self-blame and shame (Nasir et al., 2018; Brockman and Dudgeon, 2019).

Recognition of the potential impacts of transgenerational and personal trauma is central to any therapeutic initiative in working with Indigenous individuals or groups (Brockman and Dudgeon, 2019). However, evidence for the effectiveness of any specific therapy with Indigenous peoples is sadly lacking (Brockman and Dudgeon, 2019).

Having reviewed a number of available therapies, our conclusion was that compassion focused therapy (CFT) (Gilbert, 2014) looked to be a promising approach to enhance the SEWB of Indigenous peoples. CFT has been specifically designed to address issues of trauma, abuse, self-criticism and shame (Gilbert, 2014, 2017), drawing on evolutionary theory, neuroscience and attachment theory (Gilbert, 2020). Central to CFT formulation is recognition of the negative impacts of past and present social determinants in the way that we relate to ourselves, with shame and self-criticism often arising as unintended consequences of vulnerability (Gilbert, 2017, 2020). Furthermore, CFT is an inherently relational therapy. Connection and disconnection to self and others are central features of CFT (Gilbert, 2017, 2020). Although the CFT conception of connection and disconnection is not currently as all-encompassing as the Indigenous conception of SEWB, one of the primary goals of CFT is to cultivate the “safeness and soothing system” (see below), which is seen as a prerequisite for the facilitation of compassion for others and self-compassion. This CFT emphasis on connection and relationality, on the role of historical and current social determinants, and on compassion as a key antidote to shame and self-criticism (Gilbert, 2017; Kirby et al., 2017) was what led us to suggest that CFT might be a particularly good fit as a therapeutic approach in working with Aboriginal and Torres Strait Islander clients.

For present purposes, two key elements that play a prominent role in CFT therapeutic strategies should be noted. First, CFT aims to enhance clients’ awareness of their emotions (Gilbert, 2017). A key psychoeducational component of CFT is the “three systems” model of emotion (Gilbert, 2010). The CFT model suggests that, through evolution, three emotional systems have developed with specific functions to:

(1) detect threats to ourselves (the Threat System)
(2) identify, pursue and experience rewards (the Drive System)
(3) experience feelings of safeness, peacefulness, calm, and connection with others and the world (the Safeness and Soothing System).

An initial goal of CFT is to enable clients to identify which of these emotional systems are dominant. For most clients, a first step may involve developing greater awareness of the pervasive influence of their threat system. Typically, a next step is to cultivate the safeness and soothing system to enhance the sense of connection that is so fundamental to SEWB.

Second, CFT proposes that we have different innate motivational systems that determine how we experience and respond to external events, or to our own threat-related thoughts (e.g., self-criticism) and emotions (e.g., anxiety, shame) (Gilbert, 2014, 2017). For instance, if we feel threatened, then our threat motivational system tends to orient us to avoid, attack or freeze in response to external threats; or to avoid, ruminate or engage in destructive behaviors in response to negative internal experiences (emotions, thoughts). On the other hand, if our caring motivational system is dominant, then we are likely to feel motivated to treat ourselves or others with care and compassion.

It is a key aim of CFT to activate and strengthen the caring motivational system, in particular by cultivating and strengthening our “Compassionate Self,” often through a series of imagery-based practices. A particular challenge for CFT therapists is to facilitate sufficient safeness by strengthening the safeness and soothing system to enable the Compassionate Self to flourish and address typical threats such as self-criticism, shame and disconnection (Gilbert, 2017). Given the extensive history of trauma, disconnection, and grief that has been experienced by Australia’s Indigenous peoples, and the prominent experience of “shame” in Indigenous society, CFT looked to be a valuable approach in working with Indigenous clients.

Studies of the relationship between compassion and mental health have provided strong support for CFT’s central propositions. For instance, people with strong fears of compassion tend to have poor mental health (Kirby et al., 2019). Relatedly, self-criticism and lack of self-compassion are highly correlated with the experience of depression and anxiety (Van Dam et al., 2011; Werner et al., 2019), including amongst Indigenous students from Canada (Chahar Mahali et al., 2020). Furthermore, evaluation of CFT treatment programs is leading to a progressively stronger evidence base for CFT’s effectiveness (Kirby et al., 2017; Craig et al., 2020). A recent systematic review (Craig et al., 2020) has suggested that CFT is at its most efficacious when delivered in a group-based format over at least 12 h (Judge et al., 2012; Cuppage et al., 2018; Ashfield et al., 2020).

The study reported here is a pilot study of an adaptation of CFT that we call *arts-based compassion skills training* (ABCST). In ABCST, compassion skills are facilitated and channeled through the medium of visual arts, supplemented by music and story-telling. As detailed below, this visual arts-based approach (Phase 2) was only developed after a more mainstream group-based CFT approach (Phase 1) was perceived as culturally inappropriate. The aim of the study was therefore to determine whether ABCST might be an acceptable, feasible, and culturally safe way to deliver a compassion-based program derived from CFT in an Indigenous Australian context. The program has been named “arts-based compassion *skills training*” because the idea of “skills training” was seen to be more culturally acceptable than “therapy” in this context, and because the form of the program is more skills-focused than therapy-focused.

## Materials and Methods

### The Development of Arts-Based Compassion Skills Training (Phase 1 to Phase 2)

The arts-based (AB) component of ABCST was only developed after a mainstream (Phase 1) approach to group-based CFT, albeit with some cultural adaptations, appeared not to work too well with two groups of Indigenous clients in an earlier iteration of this project. Participant feedback indicated that the Phase 1 version was not perceived as culturally appropriate or culturally safe. For example, the early phases of “mainstream CFT” usually involve a strong verbal/psychoeducational component (e.g., conveying ideas derived from evolutionary theory and neuroscience). Many group members in Phase 1 found the concepts and language did not fit their way of thinking. Mainstream CFT typically makes extensive use of imagery exercises; but for our Phase 1 group members, imagery work triggered traumatic memories that were overwhelming. Similarly difficult experiences with imagery have been reported in some studies with non-Indigenous clients with traumatic backgrounds (Naismith et al., 2018).

Other Western psychotherapies such as cognitive behaviour therapy have also required significant adaptations to fit different cultural contexts (Iwamasa and Hays, 2019), so it was not entirely surprising that this direct translation of a Western psychotherapy into an Australian Indigenous context did not sit well with some group members. It therefore became necessary to re-think the approach.

Two of the Phase 1 group members gave feedback that it might be valuable to use art when working with Indigenous peoples. They suggested that focusing on art might provide more of a cultural fit, as has been suggested by other researchers working with Indigenous peoples (Jersky et al., 2016; Hammond et al., 2018). Moreover, a study from Canada (of which we were not aware at the time) had concluded that combining CFT and art might be a good fit (Williams, 2018). Accordingly, the first two authors (JBL, NR), in collaboration with a local community artist (SB), developed a six-session arts-based group approach (Phase 2), which we have termed “arts-based compassion skills training” (ABCST).

### Setting

This study of an ABCST group (Phase 2) took place in regional New South Wales, Australia in 2018. The group, specifically for Indigenous Australians, met for a 4 h session every week for 6 weeks. The sessions were facilitated by two clinical psychologists (one Indigenous) with the assistance of two local artists (one Indigenous). The group sessions took place in an Aboriginal health service. Anecdotal reports of high self-criticism and shame amongst clients and some health workers led to the proposal that the primary purpose of the groups should be to enhance self-compassion skills.

### Participants

The 10 ABCST participants were five Indigenous clients of the health service and five health professionals (4 Indigenous, 1 non-Indigenous) employed there. Participation was voluntary. Due to life circumstances, the number of participants attending each session varied from four to eight (*M* = 5.67).

The clients and the health professionals were a purposive sample nominated by the health service as appropriate for an ABCST group. Exclusion criteria were an active psychotic condition or current suicidal ideation. Whilst mental health was not formally assessed using a Western diagnostic framework due to concerns about cultural validity (Mullins and Khawaja, 2018; Brockman and Dudgeon, 2019), it was apparent that the social and emotional wellbeing of each of the clients was compromised: the experience of depression and/or anxiety appeared to be common to all.

The health professionals were included in the program as full participants to build a culturally safe space and to support clients in the event that any of them became distressed and needed to leave the room. Sharing personal stories between the health professionals and clients was not unusual in this Aboriginal health service. This sharing helps to normalize the common humanity of historical trauma and social disadvantage amongst Indigenous Australians. Only one of the five clients had a direct case management or therapy relationship with any of the five health professionals. Far from being a problem, this relationship was experienced as supportive in the group context.

### Protocol

Each session of the 6 week ABCST program consisted of three parts. The first part of the session, 20 min in duration, was led by one of the clinical psychologists, who orientated the group toward that week’s compassion topic. Some examples of weekly topics were: *What is Compassion?, Creating Safeness, the Three Systems Model and the Three Flows of Compassion (compassion for others, receiving compassion from others, self-compassion).* During this early part of the session, the psychologist engaged the group in a discussion by drawing upon participants’ life journeys and inner wisdom and exploring how their experiences related to compassion.

For the next 3 h, including morning tea and lunch, the community artists led the group with encouragement and humor in the creation their compassion art cards (approx. 20 × 10 cm). Artistic instruction and feedback was provided if requested. Music selected by the members of the group and informal story-telling were other key features of the program. An initial aim was to create a sense of group safeness in which the safeness and soothing system and creativity could flourish. If they found themselves triggered or upset, participants were encouraged to step outside the room, and take a few minutes with one of the health professionals.

The psychologists and Indigenous health professionals were full participants in this section of the program, creating their own artworks alongside the clients. Participants were provided with a range of arts materials (e.g., paints, crayons, glitter, magazines for collage, printed compassion-oriented words). On A6 size cards, these materials were used to represent the aspects of compassion most salient to the participants and the topic of the day.

The third part of the session (~30 min) was led by one of the psychologists. Participants shared the stories of their day’s artworks, drawing on personal experience to reflect on how compassion was involved in their stories. Each week finished with one of the core CFT practices, soothing-rhythm breathing, which aims to stimulate the vagus nerve and activate the parasympathetic nervous system (Gilbert, 2010, 2017).

After the end of the program, the participants’ artworks were professionally printed. The result was a boxed set of “compassion cards,” like a pack of playing cards. A set of these cards were given to each of the participants. See Table 1 for examples.

**TABLE 1:** Data analytic method: the Braun and Clarke (2006) six-phase process.

**Phase**	**Activities**
1. Familiarize with the data	In this first phase, one of the interviewer-researchers (LH) immersed herself in the data, reading and re-reading each participant’s interview to familiarize herself with the whole data set. A data management system, NVivo, was used to store and manage the extensive data. All records of the data, transcripts and process were archived. Initial reflections about potential codes/themes were documented.
2. Generate initial codes	LH generated preliminary codes, developed a coding framework to organize the codes, and kept an audit trail of code generation. NVivo was used to manage the data and create the coding framework. A second researcher (JBL—one of the group facilitators) was introduced to create researcher triangulation and enhance the credibility of the process, LH and JBL reviewed the preliminary codes against the quotes and adjusted the codes as necessary.
3. Search for themes	Researcher triangulation continued. LH and JBL wrote each code on a card. All the cards were mounted on a wall. The researchers grouped and re-grouped the cards into preliminary higher order themes. An initial tree diagram was generated to connect the themes.
4. Review themes	Researcher triangulation continued. Each theme was reviewed by LH and JBL. Key quotes were selected which illustrated each theme. The themes were reviewed and in some cases re-categorized after determining their match with the raw data. Revised tree diagrams were generated, available for the audit trail.
5. Define and name themes	Researcher triangulation continued. The key themes were defined and named by LH and JBL. They were then reviewed by JBL and a third researcher, group facilitator NR, to enable further researcher triangulation. Some minor changes to names of themes were recommended, prior to a final review by LH. Team consensus on themes was reached. A revised tree diagram was generated.
6. Produce a report	There was continued prolonged engagement with the data. A preliminary report was delivered to the funders at the conclusion of the project. There were conference presentations in first year after project completion. This article was submitted for publication in the second year, after being circulated to two of the health professionals (co-authors) for member checking.
	

### Data Collection

One to three months after the ABCST group, eight participants who attended at least three of the six sessions were contacted by telephone and invited to participate in post-program interviews. Six participants, who attended an average of 4.33 sessions were available for interview. The six participants (5 female, 1 male; *M* = 46.7 years) were two clients and four of the health professionals. Neither of the interviewed clients had a case management or therapeutic relationship with any of the health professionals. Accordingly we consider that the interview data are free from any actual or perceived conflicts of interest.

### Interview Schedule

A semi-structured interview schedule was developed to assess participants’ experience of the ABCST program. The interviews were conducted by two researchers (LH, SE), who had not taken part in the ABCST groups.

Interview questions were open-ended, focusing on the following issues:

• whether or not the program had been helpful and if so, how
• whether or not participants had felt safe
• whether or not the program had felt culturally appropriate and relevant
• what had been difficult
• what they would have wanted to be different
• whether there had been any change in their relationship with themselves or others as a result of the program
• whether their understanding of compassion had changed as a result of the program

For the health professionals, the questions addressed not only their own experience, but what they observed to be the experience of the clients. The interviews ranged between 17 and 60 min. The interviewer-researchers (LH and SE) transcribed the audio-recordings of the interviews.

### Data Analysis

**FIGURE 1:**
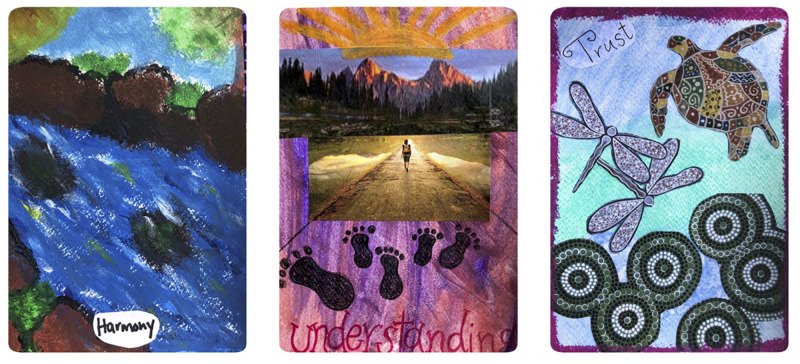
Examples of the ABCST Group’s Compassion Art Cards.

**FIGURE 2:**
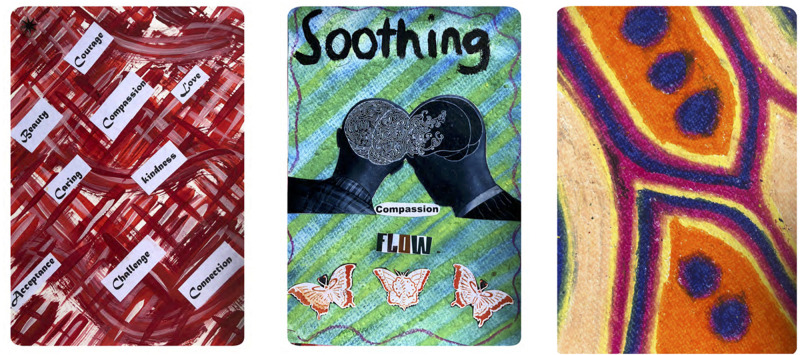
Examples of the ABCST Group’s Compassion Art Cards.

**FIGURE 3:**
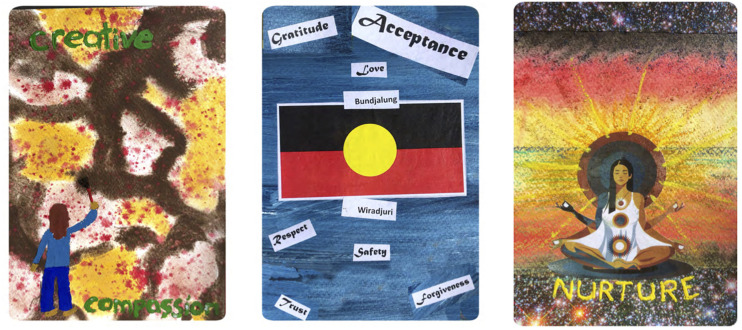
Examples of the ABCST Group’s Compassion Art Cards.

The data was subjected to thematic analysis using the qualitative analytic method described by Braun and Clarke (Braun and Clarke, 2006). They outline a six-phase process of thematic analysis, which is illustrated in Figures 1–3, alongside a description of the research activities associated with each phase. Braun and Clarke (2006) and other authors (Tong et al., 2007; Nowell et al., 2017) have identified a number of data analytic processes designed to enhance the trustworthiness and credibility of qualitative research findings. As illustrated in Table 1, the design of the current study followed many of those recommended processes (Tong et al., 2007; Nowell et al., 2017), for example: prolonged engagement with the data, researcher triangulation involving three researchers, data management strategies for complex data; extensive documentation and archiving, using a coding framework, creating an audit trail of code generation, diagramming to make sense of connections, and connecting raw data with codes and themes for credibility checks.

## Results

**FIGURE 4:**
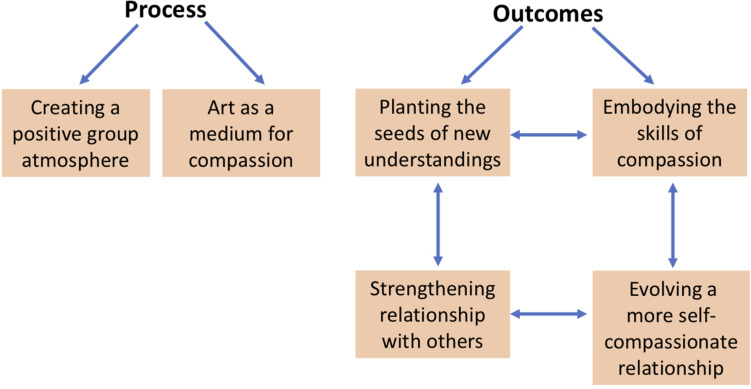
Six key themes from the Qualitative Analysis.

Two principal categories that are common to many psychotherapy studies emerged from the data: Process (of ABCST) and Outcomes. Within Process, there were two main themes: *Creating a Positive Group Atmosphere* and *Art as a Medium for Compassion* (see Figure 4). Within Outcomes, there were four main themes: *Planting the Seeds of New Understandings, Embodying the Skills of Compassion*, *Strengthening Relationships with Others*, and *Evolving a More Self-Compassionate Relationship*. Description of these themes with illustrative examples are provided below. Participant quotes are identified as Clients (C1, C2) or Health Professionals (HP1, HP2, HP3, HP4).

### Process of ABCST

#### Creating a Positive Group Atmosphere

Participants consistently remarked on the warmth, supportiveness, safeness and strength of the group atmosphere. In reference to the facilitators (clinical psychologists and artists), they used terms such as “humble,” “gentle,” “non-threatening,” “non-judgmental,” “mindful,” and “accommodating,” as well as more upbeat terms like “animated.”

A frequently noted observation was: *“there was no distinction made between facilitators and participants”* (HP2). Quite a unique feature of the 3 h art time was that the psychologists and health professionals did their own art alongside the clients, while the artists facilitated this section of the program. The participants were surprised and delighted at the non-hierarchical nature of the process. They didn’t expect that the psychologists would *“get down and dirty”* (HP2). A client remarked that *“as a group, we all participated with the cutting out and drawings and the paintings and doing the cards, the symbols, what the meanings were*… *the group dynamic was just so overwhelming”* (C1). (The client used “overwhelming” here to mean “overwhelmingly positive”).

The ABCST context of art, plus music chosen by participants and informal yarning (chat), also appeared to facilitate the group process and elevate the group atmosphere. *“Just talking, sharing stories, laughing, playing music. that was a big connection with everyone*… *through music”* (HP1).

#### Art as a Medium for Compassion

There was strong recognition that channeling CFT through the medium of art represented a cultural fit for Indigenous Australians. *“It’s relevant, especially through the art. That’s an appropriate way to teach, through art”* (HP3). *“Some wonderful cards were made from our paintings, that was very personal. That was a great resource to have. I think they [the clients] were quite blown away to see something tangible”* (HP2).

Art was seen to enhance connection and conversation. *“I was able to connect to them [clients] on a different level, through this medium, the art*… *when you’re doing something it’s much easier talking about issues that are coming up for you”* (HP2). *“If it was just set in theory, you probably would’ve lost a lot of your participants. I believe there would have been a lot more disengagement”* (HP2).

However, as one of the Indigenous staff noted, this was much more than an art class. *“The magic comes out of combining art with compassion focused therapy. It’s the way it was done*… *You could always get some artist to come in and you could always get a psychologist professor dude to come with a concept, but it is quite special how [it was] married it together”* (HP3). *“That medium was really good. It was just a winner doing it that way. Anybody black, white, or brindle could take to that way of delivering the program”* (HP2).

The HPs noted that there was a difference between discussing ideas about compassion and channeling compassion through arts-based processes. *“You don’t have to be intellectual to do a drawing or create”* (HP2). Another HP remarked: *“It’s easy to switch off when someone is telling you jargon, like big words and stuff that you don’t understand, that you need a dictionary to look at. I would say it’s pretty hard to relate to that. Rather than just what was generally getting passed around the room: people’s journeys, people’s lives, everyone’s honesty, that stuff is way richer than that other stuff. There’s a power in that”* (HP3).

Painting the cards seemed to enable participants to process their experience deeply yet safely. As one client said: *“The cards are more healing and helping me understand a little bit more*… *just looking at the painting, it just takes me into another dimension altogether”* (C1). A staff member who had previously been exposed to a more conventional verbally based CFT group contrasted the arts-based approach with her previous CFT experience, which had triggered past hurtful experiences. *“I’ve done it before, the compassion focused therapy, where it wasn’t through the art*… *I was more into the hurt I suppose. That’s all I could sort of see, a lot of hurt I’ve caused others, others have cause me*… *so it brings that stuff up for me. But this different way, I can remember there is drive, threat, and now soothing components to the compassion. Self-compassion.*… *This time I got introduced to self-compassion in a totally different way. But the other model, without the art, it was*… *abrasive”* (HP3). In short, for this Indigenous health professional, art provided a culturally safe context that seemed to be highly compatible with compassion.

### Outcomes of ABCST

The interviews with the six participants—clients and health professionals—indicated that the ABCST groups had *planted the seeds of new understandings* about compassion, and led to *embodying compassion skills*, which in turn led to a *strengthening of the relationship with others*, and a different *more compassionate relationship with themselves*. Examples from each of these categories are given below.

#### Planting the Seeds of New Understandings

Two core elements of CFT are (1) early discussion of the meanings of compassion and (2) creating a rationale for focusing on compassion, see Figure 4. In ABCST, these ideas become pictorially represented through paint, collage and printed words as illustrated in Figures 1–3. There were a number of ways in which participants’ understandings broadened as a result. One client remarked: *“I just thought compassion was just compassion when someone was dying. That’s the way I looked at it.*… *And there is more to compassion like warmth and, you know, nurturing and comfort and you know all these*… *I didn’t realize there was so many words that fit into compassion”* (C1).

Extending the idea of compassion to self-compassion was a novel concept for many in the group. As one HP noted: *“A lot of us, probably without even knowing, we display a lot of empathy and compassion towards other people, maybe we weren’t aware that we were or we weren’t displaying that to ourselves”* (HP1). Similarly, a client commented: *“I thought of compassion for other people, but not myself”* (C2).

Opening up the concept of compassion enabled participants to see that self-criticism and lack of self-compassion were common experiences amongst group members. Participants noted a possible reduction in their sense of isolation and shame as they recognized their common humanity. *“It showed to me that I am not the only person that needed self-compassion. There are a lot of people out there that need self-compassion just as much as me”* (C2). Experiencing common humanity also enhanced participants’ self-compassion: *“When you hear other people’s stories, and you kind of like ‘oh, I can relate to that’. And it’s good to know*… *that we are not the only one that is feeling like that. To hear it from somebody else, it makes you feel. a bit of relief”* (HP4).

For some members, the three systems model (the threat, drive and soothing emotional systems) had particular resonance. One of the health professionals recognized that *“I am sort of in drive and threat a lot of the time, so I have to connect more to the soothing, to centre myself a bit more, calm, decision making changes too. A lot of different things stem of that”* (HP3).

#### Embodying the Skills of Compassion

Both during group time and in-between sessions, participants noted how compassion had registered as a change in bodily experience. At one session, a client had arrived very distressed after an argument with his son but this changed once he started painting. One of the other clients observed: *“He felt really relaxed after he had done his artwork and he said ‘gee, that feels really good’. He said ‘I let my frustrations out’, but when the frustrations come out, his artwork was so lovely, so beautiful and you wouldn’t think that came from an angry man, the artwork that he ended up doing. It was completely different. The anger wasn’t there. It was more colourful and more cheerful and he said that he felt really good and relaxed after doing the painting and it made him more aware to be compassionate about himself and to be compassionate towards his son*… *it made us all feel good ‘cause we were trying to cheer him up, but he cheered his own self up with his artwork”* (C1).

Outside of sessions, clients reported using the compassion resources. One client noted about soothing-rhythm breathing: *“The breathing techniques, I have taken that on board. When I start feeling a bit of anxiety I use my breathing techniques*…. *It makes me relax instead of being so hyperactive”* (C2). Another participant particularly valued the cards of the group’s artworks, which were printed soon after the six sessions were completed. “*I put mine on my breakfast table and look at them every morning, a different card each day*… *And just reflecting back to that memory and that time sitting around the table. Like sitting around a campfire. That yarn coming up again. Yeah and that yarn is going to carry on and linger, you know, and it is going to linger and linger and linger. It is never going to go away*” (C1).

#### Strengthening Relationships With Others

For some participants, relationships with family and friends appear to have been strengthened following the program. A client reported that *“It has changed the relationship with my flatmate. We had a few arguments there before*… *but it has seemed to have helped since I have done this course*…. *Not retaliating back*… *he pays for a lot around here and does a lot for me and I suppose I took it for granted so now I am just more compassionate to the fact that he helps me a lot”* (C2). In a similar vein, one of the Indigenous health professionals remarked: *“I know it made me really look a lot more at myself and the way I do things with my children, around*… *listening to them, trying to understand them even more”* (HP1).

Interestingly, the program also seemed to enhance participants’ courage to provide feedback and create stronger boundaries in order to change the nature of some relationships which had been detrimental to their wellbeing. For instance, a client had a realization about a friend: *“I am thinking: why did I let this person walk all over me, why did I let her drink at my place, why did I let this happen? But I realized: ‘No, I let you come in, I let you spend time here. I don’t drink. You drink in my place, you had no respect for me*… *all you wanted to do was lay around, drink, watch DVDs*,… *watch movies, when I could have been doing what I wanted to do’. So now I just realize I don’t got to jump for people anymore*… *It is either my way or it is no way at all”* (C1).

Similarly, one of the health professionals changed her behavior with her family: *“I am the type of person that helped everyone in my family: financially, emotionally, physically*… *And I started saying no and I was looking out for myself. So this kind of fell into place and came in a really good time cause it kind of made me understand a little bit more about myself. It made me realise that saying no was okay, and it didn’t make me feel bad or guilty”* (HP4).

#### Evolving a More Self-Compassionate Relationship

Clients and health professionals also began to develop a more compassionate relationship with themselves: not only by creating stronger boundaries as above, but also by recognizing the importance of developing their soothing system and choosing to motivate themselves with compassion.

A health professional reported: *“I realised within the group that myself personally that I go on a lot of drive and threat. I never really go into the soothing. That was like a new awareness for me”* (HP3). The ambience of the group was such that participants were able to slip easily into mindfully reflective states: *“Sometimes silence is good. Just to sit in yourself. And then you start reflecting as well: “What’s going on here? Why am I thinking this way?”* (HP1).

A client noted that *“I pretty well didn’t have self-compassion until I went and did that course. It has changed me into giving myself more”* (C2). Compassion created motivation. She continued: “*Well usually I would just stay in bed and not do much, do nothing really, and just be depressed. But since doing the course*… *I motivate myself now. Instead of just staying in bed I get out in the garden or do some painting or do something creative just to make myself feel better.”* Moreover, “*I have actually moved house. I was living next door in a unit and I have moved here. It helped me, I suppose, make the decision to move because without compassion to myself I didn’t really care where I lived*” (C2).

In linking compassion with art, participants were also developing new skills as well as a new relationship with themselves through the program: “*I think they were surprised, as I was, at how much they actually can do paintings, given the theme that they were given. They were quite surprised with the fact that they could actually (including me) create an image”* (HP2). She continued: “*I found parts of me that I didn’t even know existed, as in a creative side that could produce something that made sense”* (HP2). It would seem that self-appreciation started to emerge in group members, alongside of self-compassion.

## Discussion

As far as we are aware, this is the first study which has trialed CFT with Indigenous clients. After unpromising beginnings with conventional verbally oriented CFT groups, the transition to arts-based CFT groups seemed to provide a much stronger cultural fit. The combination of creating a positive group atmosphere and channeling CFT through the medium of art led to new understandings of compassion and embodiment of compassion skills. In turn, participants reported that these changes led to greater self-compassion and compassion for others. Although this was a pilot study with a small group of 10 people and six follow-up interviews, what was notable was the complete contrast between participants’ experiences of ABCST and the previous verbally oriented CFT groups.

A key issue raised by the present study is the need to make adaptations to western psychotherapies for different cultural contexts (Iwamasa and Hays, 2019). Modern Western psychotherapies such as CBT, ACT, and CFT are largely based on a set of evidence-based psychological principles that are considered to be generally applicable to human suffering. However, it is also well recognized that for these therapies to be effective in Indigenous (Nelson et al., 2014; Toombs et al., 2020) or other cultural contexts (Rathod and Kingdon, 2009; Iwamasa and Hays, 2019), they need to be culturally responsive. Moreover, in order to be culturally responsive, community engagement processes such as co-design workshops and reference groups with key stakeholders over an extended period of time are required (Singer et al., 2015; Toombs et al., 2020).

In the present case, this CFT project had pilot funding for just 1 year. This tight timeline precluded the kind of more extended co-design or community involvement processes that would have been desirable. Our solution to the tight timeline was to pilot a preliminary four session CFT trial with Indigenous health professionals, using the verbally oriented format typically reported in non-Indigenous contexts. Although the Indigenous health professional group voiced some reservations about this approach, we retained this format in the absence of any recognized alternatives, while making adaptations consistent with their feedback. However, after the first set of groups, it became clear that that format did not meet the participants’ needs.

As reported here, participants’ experiences with ABCST were radically different. There were strong themes of safeness and connection. They experienced the facilitators and each other as supportive, gentle, non-threatening, non-judgmental, and accommodating. They reported strong connections with one another through the sharing of stories, music and art. As well as connecting with one another, they also experienced greater care/positive connection with themselves, and experienced the medium of art as enhancing their soothing system. Connection is central to the concept of SEWB. Though the links between the processes of ABCST and SEWB have yet to be fully articulated, the importance CFT places on the safeness and soothing system in helping to balance the threat and drive systems, and CFT’s relational focus in facilitating connection suggests ABCST’s promise as a partner in initiatives to enhance SEWB for Indigenous Australians.

We believe there are a number of reasons why art was found to be a natural partner for CFT in Indigenous contexts. First, art is well-recognized as a core part of Indigenous Australian culture and represented a natural cultural fit (Jersky et al., 2016), even for the majority of participants who had no pretensions to be artists.

Second, the nature of art is that it is a slow, absorbing process. As an activity, art would seem to have a natural affinity with the safeness and soothing system (Gilbert, 2010, 2017), drawing participants into a creative realm where their imagination can flourish. Contrast this with verbally oriented groups featuring new ways of thinking that may be difficult to grasp conceptually. It is not hard to see that for some participants—Indigenous or non-Indigenous - such groups will trigger their threat systems. One client from a related study who attended a conventional CFT group and a 1 day ABCST workshop summarized the difference this way: *“Instead of sitting in a room with someone that you sort of didn’t know, someone you maybe thought was judging you*… *You are not in that headspace of guilt, the headspace of shame, you are in the headspace of*… *things that are joyful in your life*… *Things that you are proud of. Things that you want in your life. For me that sort of helped a lot.”* In CFT terms, it makes sense that when people are grounded in their soothing systems, it is far easier for them to develop their Compassionate Self than when they are sitting in their threat systems.

Third, we re-emphasize the comment of one of the health professionals, *“the magic come out of combining art with compassion focused therapy,”* reinforcing the view that art and compassion are natural partners. By its very nature, art may often facilitate a focused, mindful state; when this is combined with the subject matter of compassion, both “wings” of the mindfulness-compassion “bird” are facilitated (Williams, 2018). An observation which we hope to follow up in future studies is that our group members appeared to experience far less of the fears, blocks and resistances to compassion which have been reported as major hurdles to be overcome in more verbally based CFT groups (Gilbert, 2010). One hypothesis is that ABCST primes the soothing system, allowing compassion to slip past the usual resistances.

Alongside of *Art as a Medium for Compassion*, the other key process variable that emerged from the data was *Creating a Positive Group Atmosphere.* Central to CFT is the idea of “safeness” (Gilbert, 2017); therapy should provide a secure base in order to activate and strengthen the soothing system. Our aim was to build in a sense of safeness from the start of the ABCST group. Group feedback suggests that this was successfully achieved. Some of the key elements were: the safe space created through the personal local knowledge of the Indigenous health professionals; the facilitation skills of the clinical psychologists and artists; the absence of hierarchy; the sense of enjoyment through art, music, story-telling and humor; the comfort of spending 4 h together including the sharing of lunch; the opportunity to admire each other’s work; and to hear the stories associated with each week’s painting.

A notable feature of the group was the spontaneous informal conversations which emerged. Some were clearly focused on the topic of compassion, and how it manifested, or didn’t, in participants’ lives. The character of these conversations was quite different from what might happen in a conventional CFT group. They were spontaneous and informal, yet there was often deep personal sharing—sometimes in pairs, or in threes or fours, or sometimes in the whole group. No-one was put on the spot; people shared their experience if they felt like it.

From a CFT perspective, participants had positive experiences of the three flows of compassion: giving compassion to others, experiencing the compassion of others, and experiencing self-compassion. Consistent with the findings from a recent qualitative study of group CFT treatment for complex PTSD (Ashfield et al., 2020), the positive group atmosphere and receiving compassion from others appear to have been two important elements of the present program.

Participants reported positive outcomes from the program, both in terms of their understanding (*Planting the Seeds of Compassion*) and their skills (*Embodying the Skills of Compassion*). These skills manifested both in their relationship with others and with themselves.

What was it about arts processes that may have been so effective in facilitating compassion? It was notable how compassion concepts became easily integrated into their art pictorially and through inclusion of words associated with compassion (see Figures 1–3). During the 3 h of art, participants became absorbed in their artworks physically, emotionally, cognitively and behaviorally. They reported that they were more easily able to take on board the concepts: *“This different way, I can remember there is drive, threat and now soothing components to the compassion.”* This was in contrast to: *“If it was just set in theory, you probably would have lost a lot of your participants.”*

Epstein (1994, 2014), Teasdale and Barnard (1993); Teasdale (1999), and other dual process theorists (Power and Dalgleish, 2008) have suggested that there is a fundamental distinction between two types of information processing system: Epstein termed these a “rational system” and an emotionally driven “experiential system” (Epstein, 1994, 2014). Teasdale and Barnard referred to a “propositional code” and an “implicational code” (Teasdale and Barnard, 1993; Teasdale, 1999). On the one hand, they suggest that there is a rational-verbal-propositional system that is analytic and reason-oriented, that has no emotional content or sensory elements, and that represents “head level intellectual belief.” This is contrasted with an experiential/implicational system that encodes information schematically through imagery and metaphor (Teasdale and Barnard, 1993; Teasdale, 1999; Hackmann et al., 2011), is closely tied to emotion and bodily sensation, is essentially non-rational and automatic, and has a felt sense of “rightness” (“heart level emotional belief”). We hypothesize that ABCST taps directly into the experiential/implicational system, in a similar way to other forms of imagery (Hackmann et al., 2011); and that the arts-based groups were therefore experienced as far more compelling, memorable and embodied compared with our previous CST groups which were rather more verbal-propositional and intellectualized in the way that the concepts of compassion were conveyed.

This is a small-scale pilot study, and as such, there are a number of limitations. First, there were 10 people in the group and only six of the 10 were interviewed. Two of these were clients, four were health professionals. We did not have access to personal histories or backgrounds of participants. The extent to which they are representative of Indigenous peoples in their own community, or in other communities, is unknown, as too is the extent to which the results might generalize to other contexts and other clients. Second, the interview data were obtained from interviews 1–3 months after the end of the program. It is not known whether or how any positive effects of the program persisted in the longer term. Third, there were no objective measures of SEWB, or compassion measures pre- and post-workshops to gauge the extent of any changes. Fourth, we have yet to determine the most important components of ABCST or the optimum length of such a program. Some core CFT practices were included in ABCST (soothing-rhythm breathing, the three systems model); others were largely implicit (e.g., “it’s not your fault”) or omitted (e.g., individual formulation, compassionate visual imagery, addressing fears, blocks and resistances). Some elements of ABCST (e.g., visual arts, art-focused story-telling, music, informal conversations, cultural practices such as acknowledgment of country) were included *de novo*. Future ABCST studies should examine which components should be included to yield the greatest benefits.

The study raises a number of other interesting questions. For instance, would other forms of art (e.g., music, or traditional Aboriginal art forms such as weaving or dancing) be equally suitable vehicles for cultivating compassion? Would visual arts or other arts work equally well with non-Indigenous clients? Should different ways of delivering group-based compassion skills training be compared to determine whether different strategies might work best in different contexts? For instance, our prior experience was that conventional CFT imagery practices were triggering for Indigenous clients; arts-based CST delivery seemed safer and more benign. Can ABCST be extended so that the processes are more explicitly oriented not just toward compassion and connection to self and others, but also toward some of the other SEWB domains (e.g., country, spirituality, culture)?

## Conclusion

In conclusion, cultural relevance is central to effectiveness of any therapy in the Indigenous space. By listening to community feedback, we changed our CFT approach from more conventional verbal-based CFT groups to CST delivered through the medium of visual arts. The contrast in participant experience was immediately apparent. Arts-based CST looks to be a more promising and efficacious mode of delivery for Indigenous clients, which appears consistent with the SEWB conception of illness and health. Future studies with larger numbers of clients in various contexts, ideally with some objective measures alongside of self-report interview data, will determine to what extent the findings from the present study may generalize to other contexts.

## Data Availability Statement

The raw data supporting the conclusions of this article will be made available by the authors, without undue reservation, to any qualified researcher.

## Ethics Statement

The studies involving human participants were reviewed and approved by the Aboriginal Medical Council Ethics Committee and the University of Sydney Human Ethics Committee. The patients/participants provided their written informed consent to participate in this study.

## Author Contributions

JB-L, NR, KL, SB, and PG were all involved in the original design of the project, and all have either contributed to written sections or commented on the drafts of the manuscript. JB-L, NR, and SB were involved in the day-to-day design and running of the arts-based compassion focused therapy groups. LH and SE were interviewers and undertook qualitative data analyses; also commented on the manuscript. GC, DO’C, and SK made contributions to recruitment and support of the Aboriginal and Torres Strait Islander participants in the project. DO’C and SK supported the clients and contributed content to the arts-based workshops. All authors have contributed materially to this manuscript.

## Conflict of Interest

The authors declare that the research was conducted in the absence of any commercial or financial relationships that could be construed as a potential conflict of interest.
